# Management of Severe Pain in a Case of Sensory Guillain-Barre Syndrome

**DOI:** 10.7759/cureus.64432

**Published:** 2024-07-12

**Authors:** Joane Titus, Bernard Sarmiento, Roger Crouse

**Affiliations:** 1 Medicine, University of Central Florida College of Medicine, Orlando, USA; 2 Neurology, University of Central Florida College of Medicine, Orlando, USA; 3 Internal Medicine, University of Central Florida Hospital Corporation of America (HCA) Healthcare Graduate Medical Education (GME), Orlando, USA

**Keywords:** therapeutic plasmapheresis, acute inflammatory demyelinating polyradiculoneuropathy, multi-modal pain management, sensory gbs, pain refractory to treatment

## Abstract

Guillain-Barre syndrome (GBS) is an acute post-infectious polyradiculoneuropathy characterized by autoantibodies targeting host antigens, resulting in nerve fiber demyelination and axonal degeneration. While symmetric ascending weakness is typical, neuropathic pain is a common yet variable manifestation. We present a case of a 52-year-old man with progressive bilateral leg weakness and severe neuropathic pain following a flu-like illness. Despite conventional analgesics, his pain persisted, necessitating a unique pain management approach.

The patient's examination revealed hyporeflexia and sensory deficits consistent with GBS. Diagnostic workup, including lumbar puncture, showed albuminocytologic dissociation. Plasma exchange therapy was initiated, but severe nocturnal neuropathic pain persisted, exacerbating during treatment. Conventional pain medications were ineffective, prompting a multimodal approach.

Combining hydromorphone and lorazepam provided significant pain relief, enabling completion of plasmapheresis sessions. This regimen, supplemented with gabapentin, proved effective in managing both GBS-associated and treatment-induced pain.

This case underscores the debilitating nature of GBS-related pain and the importance of tailored pain management strategies. While conventional agents may fail, a multimodal approach, including opioids and adjunctive medications, can offer relief, facilitating essential treatments like plasmapheresis. Careful monitoring is imperative to mitigate risks associated with potent analgesics. Our experience contributes to the armamentarium for managing GBS-related pain, emphasizing individualized care to improve patient outcomes.

## Introduction

Guillain-Barre syndrome (GBS) is an acute post-infectious polyradiculoneuropathy where cross-reactive autoantibodies target host antigens, resulting in demyelination and axonal degeneration of motor, sensory, and autonomic nerve fibers [1,2]. The characteristic presentation is a symmetric, ascending weakness with decreased or absent deep tendon reflexes. Acute inflammatory demyelinating polyneuropathy (AIDP) is a phenotype within the GBS spectrum [1].

GBS occurs when cross-reactive autoantibodies, which form usually after a gastrointestinal or respiratory infection, target host axonal antigens of peripheral myelin or Schwann cells. This attack leads to immune-mediated segmental demyelination and axonal degeneration of motor, sensory, and autonomic nerve fibers [2]. This results in the characteristic symmetric and ascending flaccid paralysis. Autonomic dysfunction can present in the form of arrhythmias, ileus, and the feared respiratory compromise. In addition to other manifestations, demyelination of peripheral and autonomic nerve fibers can lead to severe neuropathic, radicular, and/or musculoskeletal pain. Pain is a common but variable manifestation affecting 55-89% of patients [3]. Such pain can precede the ascending weakness and persist long into recovery.

## Case presentation

A 52-year-old gentleman presented to the emergency department with three days of worsening and progressively ascending bilateral leg weakness, accompanied by lower leg myalgias and paresthesia in distal toes. Three weeks prior, he was diagnosed with a flu-like illness after developing a fever, cough, and myalgias. He first noticed muscle soreness in his feet, which then progressed to severe lower leg weakness. The patient had difficulty getting out of bed and ambulating due to the weakness in his feet. The weakness then ascended bilaterally to his ankles and then to his thighs.

On examination, the patient had a visibly anxious affect with hyporeflexia of the biceps, triceps, Achilles, and patellar reflexes. Additionally, there was loss of vibratory, temperature, and pinprick sensation in his lower extremities up to the level of the medial malleolus bilaterally. Laboratory tests, including blood count and metabolic panel, were unremarkable as well as CT/MRI head/spine imaging. Lumbar puncture showed an albuminocytologic dissociation with an elevated protein of 167 mg/dL, zero white blood cells, one red blood cell, and normal glucose.

The patient received six sessions of plasma exchange therapy. Throughout the course of his two-week hospitalization, he experienced severe, debilitating neuropathic pain described as burning in his legs, arms, shoulders, and lower back that was predominantly nocturnal and associated with the onset of plasmapheresis treatment. Initial pain management included acetaminophen, ketorolac, methocarbamol, gabapentin, and lidocaine patches.

Despite this, the tear-inducing neuropathic pain that peaked around 1-3 AM led to sleepless nights, described as “I am burning alive.” Further pain management included trials of pregabalin, carbamazepine, duloxetine, oxycodone, and baclofen. The most effective pain medication regimen that helped with plasmapheresis-associated and nocturnal episodes of pain was a combination of hydromorphone 0.5 mg with lorazepam 1 mg that was given together every four hours. The patient's pain management regimen eventually provided him with relief (Table 1).

**Table 1:** Pain Medications, Dosing, and the Level of Pain Relief *Pain Relief is measured by the change in the patient’s morning pain scale (1-10) from prior to starting that medication: with no pain relief (change by 0), minimal pain relief (change by 1), moderate pain relief (change by 2-3), and significant pain relief (change by 4+). SNRI: serotonin and norepinephrine reuptake inhibitor, NSAID: nonsteroidal anti-inflammatory drug.

Drug	Medication Class	Dosage Used	Level of Pain Relief*
Gabapentin	Anticonvulsant	900 mg TID	Minimal
Carbamazepine	Anticonvulsant	200 mg TID	None
Pregabalin	Anticonvulsant	75 mg BID	None
Duloxetine	SNRI	30 mg QHS	None
Ketorolac	NSAID	15 mg Q8H	None
Acetaminophen	Non-opioid analgesic	1000 mg Q6H	None
Methocarbamol	Skeletal muscle relaxant	1000 mg Q8H	None
Baclofen	Skeletal muscle relaxant	20 mg TID	Minimal
Lidocaine 4% patch	Local anesthetic	Two patches BID	None
Oxycodone	Opioid	10 mg TID	Minimal
Hydromorphone	Opioid	0.5 mg Q4H	Moderate
Lorazepam	Benzodiazepine	1 mg Q4H	Moderate

## Discussion

GBS can be a debilitating AIDP. The primary focus of GBS treatment is to address the underlying immune response and pain management. Refractory pain in GBS can lead to treatment dilemmas since conventional medical treatments can prove to be ineffective. Characteristic pain in GBS can be multifaceted, presenting in various forms. The pathophysiology can include neuropathic pain, which is a result of nerve damage leading to a burning, stabbing pain, along with musculoskeletal pain. The approach to pain management is often multimodal, including medications and supportive therapies such as analgesics and opioids for severe pain. Opioids are reserved for second- and third-line treatments given the risk of dependency. Neuropathic treatment medications include gabapentin, pregabalin, and tricyclic antidepressants. Nonpharmacological treatments include physical therapy (PT) and transcutaneous electrical nerve stimulation (TENS).

In this atypical GBS case, muscle and radicular pain preceded weakness, which is found in only one-third of patients [4]. The extremely severe nocturnal neuropathic pain was unrelieved with many of the first- and second-line agents used for GBS-related pain. When pain is refractory, such as in our patient, the pathophysiology can include worsening severe nerve damage secondary to extensive demyelination and axonal damage, and variances in metabolic rates affecting efficacy. Advanced pharmacological therapies include intravenous immunoglobulin (IVIG) and plasmapheresis, which are mainstays of GBS treatment.

Pain management is a key component of supportive care in GBS patients, especially when compounded by a psychological component due to anxiety. Gabapentin and carbamazepine have proven efficacy in neuropathic pain in GBS patients compared to placebo [5], yet carbamazepine provided no relief. In fact, first-line pain medication options usually do not provide adequate relief, as evidenced by the fact that 75% of GBS patients require oral or parenteral opioids [5-8]. Other management options can include interventional procedures such as nerve blocks or epidural analgesia, which were not used in our patient. Given the severe mental stressor of pain in such a condition, psychological support is encouraged through the use of cognitive-behavioral therapy (CBT) to help patients cope with chronic pain. The effective pain medication regimen is noted in (Table 2). This regimen provided moderate to significant pain relief when used together, especially during plasmapheresis sessions, and also helped reduce anxiety.

**Table 2:** Effective Pain Medication Regimen The table has been created by the authors with regard to the patient's treatment regimen.

Drug	Medication Class	Dosage Used
Gabapentin	Anticonvulsant	900 mg three times daily
Hydromorphone	Opioid	0.5 mg every 4 hours
Lorazepam	Benzodiazepine	1 mg every 4 hours

Respiratory status monitoring, such as negative inspiratory force (NIF) checks, is critical for GBS patients, especially when receiving a cocktail regimen of combined opioids and benzodiazepines [5,9].

The complexity of this disorder requires a multidisciplinary approach with neurologists, pain specialists, physical therapists, and psychologists. Regular reassessment of pain and progression of GBS is necessary. This case highlights how excruciating GBS-related pain can be and how critical pain management is when providing supportive care for GBS patients. Although GBS pain is common, with 55-89% of GBS patients reporting pain in the acute setting, its manifestations are variable [3]. Studies have shown efficacy in agents such as gabapentin and carbamazepine for GBS neuropathic pain compared to placebo [4]. However, the pain in this case was unrelieved by many of the first- and second-line agents.

First-line pain medications usually do not provide adequate relief, as evidenced by the fact that 75% of GBS patients require oral or parenteral opioids [5]. The hydromorphone 0.5 mg and lorazepam 1 mg cocktail, with standing gabapentin 900 mg three times daily, provided significant pain relief when used together and even allowed for the completion of the patient’s plasmapheresis sessions. It is critical to monitor respiratory status in GBS patients, especially in patients receiving such a cocktail. By outlining the above strategy, we hope to provide another tool in managing GBS-related pain.

## Conclusions

Pain refractory to treatment in GBS poses a significant challenge, requiring innovative and individualized approaches to management. While conventional treatments provide relief for many, a subset of patients needs more intensive and multidisciplinary strategies to manage their pain effectively. Ongoing research and clinical advancements hold promise for better understanding and treating this debilitating aspect of GBS. By providing a case of acute inpatient management of GBS-related pain, we hope to summarize some of the available options for neuropathic pain relief.
